# Serine/Threonine Kinase 4 Deficiency Caused by a Potential Novel c.248del, p.Pro83Leufs∗3 Mutation: A Case Report

**DOI:** 10.1155/crii/8573882

**Published:** 2026-08-14

**Authors:** Hamidreza Ashayeri, Shabnam Eskandarzadeh, Samin Alavi, Mohammad Saberi, Azadeh Reshadmanesh

**Affiliations:** ^1^ Research Center for Evidence-Based Medicine, Iranian EBM Centre: A JBI Centre of Excellence, Faculty of Medicine, Tabriz University of Medical Sciences, Tabriz, Iran, tbzmed.ac.ir; ^2^ Department of Pediatrics, Pediatric Health Research Center, Tabriz University of Medical Sciences, Tabriz, Iran, tbzmed.ac.ir; ^3^ Pediatric Congenital Hematologic Disorders Research Center, Research Institute for Children’s Health, Shahid Beheshti University of Medical Sciences, Tehran, Iran, sbmu.ac.ir; ^4^ Department of Medical Genetics, School of Medicine, Tehran University of Medical Sciences, Tehran, Iran, tums.ac.ir; ^5^ Department of Medical Biotechnology, Faculty of Medical Sciences, Tarbiat Modares University, Tehran, Iran, modares.ac.ir

**Keywords:** combined immunodeficiency, inborn errors of immunity, STK4 deficiency, T-cell deficiency

## Abstract

**Background:**

Serine/threonine kinase 4 (STK4) deficiency is one of the rare causes of combined immunodeficiency (CID). In this study, we report a novel mutation causing STK4 deficiency, which presents with chronic pyelonephritis, an uncommon presentation.

**Case Presentation:**

A 10‐year‐old female with an incidental mass in the kidney extending to the liver, night sweats, and fever was presented. To reduce the mass effect and rule out the possibility of malignancy, the patient underwent a nephrectomy, and a biopsy revealed chronic pyelonephritis. After surgery, due to the persistent leukopenia, the chance of an inborn error of immunity was considered. The patient was referred to the immunology clinic.

**Investigations:**

Immunophenotyping revealed low levels of CD3 and CD4. The antibody titer did not show a significant rise after receiving diphtheria and tetanus vaccines. Whole‐exome sequencing was performed, and a novel homozygous c.248del, p.Pro83Leufs ^∗^3 variant was reported in the *STK4* gene. The presence of this variant in parents was also confirmed with Sanger sequencing.

**Conclusion:**

STK4 deficiency is a rare cause of CID characterized by susceptibility to recurrent infections. Our case shows immune dysfunction manifested as a complex inflammatory syndrome, featuring an abdominal mass that mimics malignancy, expanding the recognized clinical spectrum of this condition.

**Highlights**

• Patients with serine/threonine kinase 4 (STK4) deficiency can experience recurrent urinary tract infections, resulting in chronic pyelonephritis alongside upper respiratory infections.
• The c.248del is a novel variant in the *STK4* gene that potentially leads to STK4 deficiency.
• Increased IgE levels may be present in STK4 deficiency.
• Patients with STK4 deficiency may have an impaired response to vaccines.

## 1. Introduction

Serine/threonine kinase 4 (STK4) has roles in T‐cell coordination and resistance to oxidative stress, and its deficiency can lead to lymphopenia and combined immunodeficiency (CID) [1, 2]. According to the OMIM database, STK4 (OMIM: 604965) deficiency is an autosomal recessive CID (OMIM: 614868) characterized by progressive T‐cell lymphopenia, recurrent bacterial, viral, and fungal infections, atopic manifestations, susceptibility to autoimmune disease, and Epstein‐Barr virus (EBV)‐associated lymphoma, typically presenting in childhood [1, 3, 4]. We present a 10‐year‐old girl with an abdominal mass, diagnosed with CID due to a novel mutation causing STK4 deficiency.

## 2. Case Presentation

A 10‐year‐old female patient was present at the pediatric clinic with a complaint of an incidental abdominal mass. The patient also complained of dysuria, right flank pain, fatigue, and night sweats. The patient’s parents are first cousins. However, the familial history of genetic disorders was negative. The patient had a history of recurrent urinary tract infections, pneumonia, and acute otitis media since she was 7. The patient had no history of eczema, atopia, allergy, or recent weight loss.

The patient had a body temperature of 38.7°C, a heart rate of 94, and a blood pressure of 120/85. The patient had no history of growth or developmental abnormalities, weighed 42 kg (86^th^ percentile based on Centers for Disease Control and Prevention (CDC) weight‐for‐age, for girls, 2–20 years of age), and was 147 cm (89^th^ percentile based on CDC stature‐for‐age, for girls, 2–20 years of age). On physical examination, the patient had hepatosplenomegaly and inguinal lymphadenopathy, but no rash or dysmorphism was observed.

**Figure 1:**
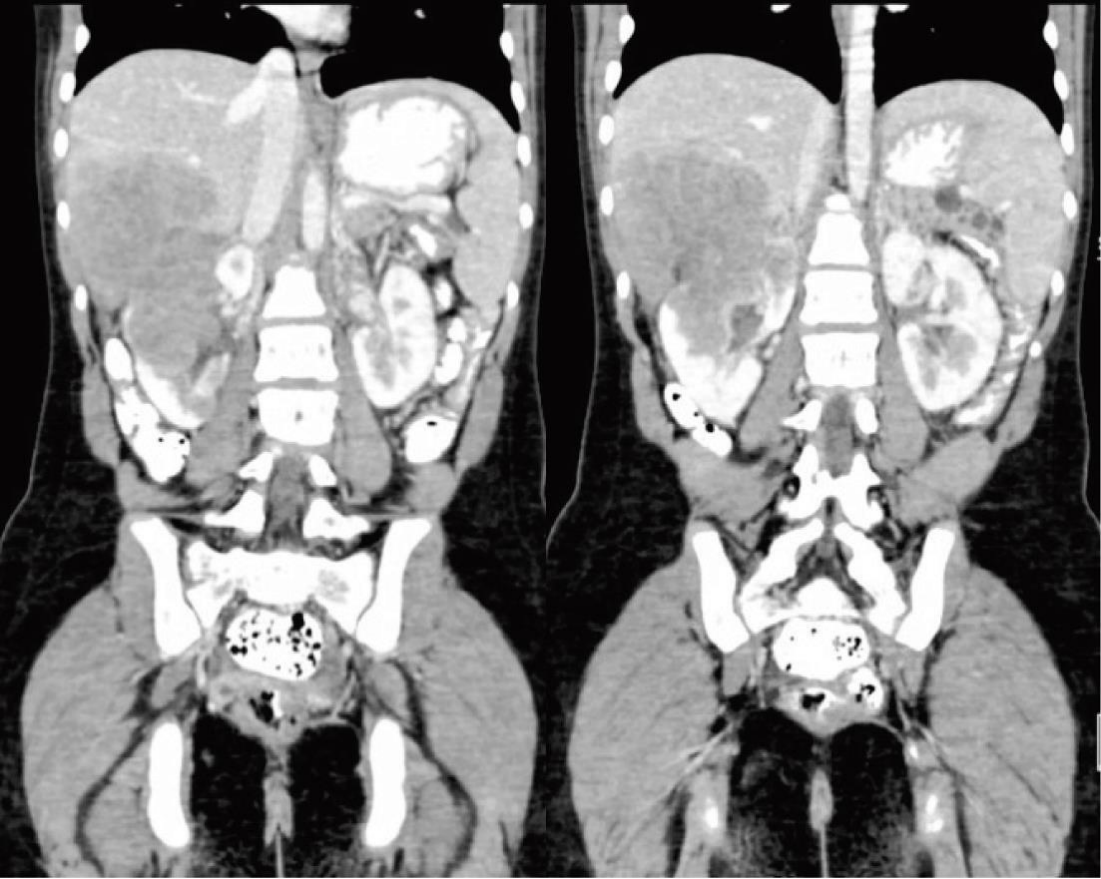
Abdominal CT of the patient reveals a heterogeneous mass in the right kidney and liver.

To assess the abdominal mass, the patient underwent a radiologic study. The abdominal US revealed a 99 mm × 80 mm × 60 mm hypoechoic‐heterogeneous mass in the right kidney with an extension to the liver, with the presence of multiple lymphadenopathies. The mass has been reported to distort the normal kidney parenchyma. The patient’s abdominal and pelvic CT revealed a large hypodense infiltrative mass with calcified foci in the right kidney that extends into the 6^th^ hepatic segment. Also, there were conglomerated lymph nodes in the aortocaval region. Figure 1 presents the patient’s abdominal CT scan. The patient’s laboratory results revealed a hemoglobin level of 12.1 g/dL (normal range: 11.5–15.5), creatinine of 0.9 mg/dL (normal range: <1.4), erythrocyte sedimentation rate (ESR) of 60 mm/h (normal: <10), with a lactate dehydrogenase (LDH) of 178 U/L (normal range: 140−350), uric acid of 3 mg/dL (normal range: 2.5–5.5), and calcium of 9.5 mg/dL (normal range: 8.5–10.5). Many white blood cells and bacteria, 5–10 red blood cells, 2+ proteinuria, and positive nitrites were reported in urine analysis (U/A) with a pH of 6 and a specific gravity of 1.010. The patient’s urine culture (U/C) was positive for *Escherichia coli* (>10^5^ CFU/mL).

To reduce the mass effect and rule out the possibility of a malignancy (especially Wilms tumor), the patient underwent a right nephrectomy and lymph node resection. The biopsy revealed sclerosed glomeruli, interstitial tissue, and sclerosis of blood vessel walls along normal‐appearing glomeruli. There were also foci of ischemic necrosis, calcification, and thrombosis in the blood vessels. The interstitial tissue was infiltrated with mononuclear cells and foamy macrophages. The lymph nodes were reactive. The IHC study of tissue was positive for CD45 and Vimentin (only for vascular structures) and was very rarely positive for Ki‐67; it was negative for CD99, Desmin, NSE, WT1, and Synaptophysin. The result of the biopsy and IHC excluded malignancies and was suggestive of chronic pyelonephritis.

Due to a history of recurrent pneumonia and otitis media, leukopenia, and diagnosis of chronic pyelonephritis, the patient underwent an immunologic assessment for the possibility of an inborn error of immunity. To measure the patient’s T‐cell‐dependent B‐cell response against antigens, baseline diphtheria Ab (IgG) and tetanus Ab (IgG) were measured, and the patient was then vaccinated against these agents. Four weeks later, the patient’s antibody levels for diphtheria and tetanus were repeated and showed no significant increase. The lymphocyte transformation (LTT) test was also performed to assess T‐lymphocyte function. Lymphocyte immunophenotyping was performed by flow cytometry using a MACSQuant cytometer (Miltenyi Biotec, Auburn, CA, USA). The markers analyzed included CD3, CD4, and CD8 for T‐cell subsets, CD20 for B cells, and CD16 and CD56 for natural killer (NK) cell populations. The patients’ lab results, LTT, and immunophenotyping are presented in Table 1.

**Table 1:** Laboratory results and immunophenotyping of the patient.

Test	June‐10	June‐29	Reference range
White blood cell	4790	3400	5000–13,000/µL
Neutrophil	3580	2200	1500–8000/µL
Lymphocyte	660	400	1200–6600/µL
Eosinophil	100	200	50–200/µL
Hemoglobin	11.8	13.7	11.5–15.5 g/dL
Platelets	325,000	245,000	170,000–450,000/µL
C‐reactive protein	10	7	<10 mg/L
Diphtheria Ab (IgG)	0.014	0.07	<0.01: no protection 0.01−0.1: no reliable protection >0.1 reliable protection
Tetanus Ab (IgG)	0.091	0.16	<0.1 basic immunization recommended 0.1–1: to be controlled after 1–2 years 1–5: to be controlled after 2–4 years >5: to be controlled after 4–8 years
IgG	1708	1615	698–1560 mg/dL
IgA	133	138	53–204 mg/dL
IgM	61	50	31–179 mg/dL
IgE	409.62	504	0–280 IU/mL
NBT	88%	100%	>90%
CD3^+^ (absolute count)	172	165	1239–2611
CD4^+^ (absolute count)	109	78	646–1515
CD8^+^ (absolute count)	92	97	365–945
CD4/CD8	1.19	0.81	1.18−2.65
CD20^+^ (absolute count)	101	114	200–2000
CD16^+^ (absolute count)	50	36	90–590
CD56^+^ (absolute count)	84	111	90–590
CD16^+^CD56^+^ (absolute count)	17	6	120–483
LTT‐PHA (control’s SI)	—	5 (8)	≥3
LTT‐BCG (control’s SI)	—	2.6 (3.9)	≥2.5

Lymphopenia, elevated IgE levels, no rise in diphtheria and tetanus Ab levels, and altered lymphocyte subsets characterized by decreased CD3^+^, CD4^+^, and CD20^+^ populations prompted consideration of CID. Following HIV exclusion by PCR, whole‐exome sequencing was performed to establish a definitive diagnosis of CID and identify potential causative variants. Whole‐exome sequencing identified a novel homozygous frameshift variant in the *STK4* gene, NM_006282.5: c.248del, p.Pro83Leufs ^∗^3, which strongly supported a diagnosis of STK4 deficiency. Antibodies against EBV and CMV were negative.

**Figure 2:**
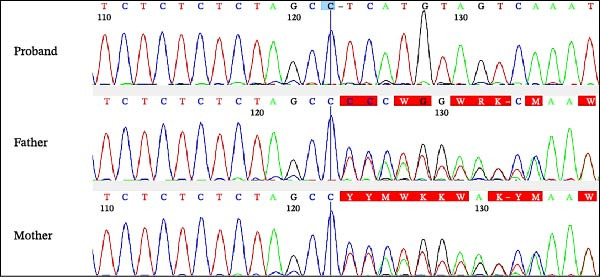
Sanger sequencing result.

Subsequently, Sanger sequencing was performed to confirm the presence of this variant in the patient and to conduct segregation analysis of the parents (Table 2). The results revealed that both parents were heterozygous carriers of the identified variant (Figure 2). Thus, confirming the autosomal recessive mode of inheritance for the STK4 deficiency. Due to the lack of resources, we could not measure mRNA levels or the effects of the mutation on the protein. The patient has received monthly IVIg and prophylactic antibodies since then and was subsequently referred for bone marrow transplantation with a possible diagnosis of STK4 deficiency. During the past year, the frequency of upper respiratory and urinary tract infections declined.

**Table 2:** Primer sets used for Sanger sequencing and the PCR thermal conditions.

Forward primer sequence	ATTTTGCATTCTTATCAGGAGGTTGTCC			
Reverse primer sequence	AGGGGAAATCTGACAGTCGTCTCT			
	Stage	Temperature (°)	Time	Cycles
PCR thermal condition	Initial denaturation	95	5 min	1
	Denaturation	95	20 s	35
	Annealing	62	20 s	35
	Extension	72	50 s	35
	Final extension	72	5 min	1

## 3. Discussion

*STK4* is proposed to play a role in T‐cell trafficking, proliferation, and differentiation [5]. Also, *STK4* is found to interact with multiple transcription factors in T cells, causing an immunoregulatory effect [6]. The age of presentation differs between the studies, and severe forms of disease are reported to be diagnosed in early years of life; however, a cohort of 15 patients reported disease development between 6 and 248 months, with a median of 6 years and 8 months [2, 7]. Due to genetic, epigenetic, and environmental factors, the age of manifestation may vary. Abolnezhadian et al. [8] report a case of identical twins with STK4 mutations, where one twin showed clinical manifestations 2 years after the other. Because of the disease’s rarity, there is also a delay in diagnosis, and the median age of diagnosis was 7 years and 5 months [2].

STK4 deficiency makes patients susceptible to *Streptococcus pneumoniae*, *Haemophilus influenzae*, EBV, human herpesvirus, human papillomavirus, and Varicella‐zoster virus [2, 9]. Bronchiectasis, autoimmune cytopenia, salt‐wasting tubulopathies, and atopic dermatitis are reported in patients with STK4 deficiency [2]. However, some cases have been reported with milder and atypical presentations [10]. Our case had severe T‐cell lymphopenia (<500/µL), manifested by chronic pyelonephritis, which is atypical of the classic STK4 deficiency phenotype.

The kidney mass with extrarenal extension suggested a renal malignancy; however, laboratory results, kidney biopsy, and IHC favored a diagnosis of chronic pyelonephritis due to recurrent UTI with reactive lymphadenopathy. The most probable type of chronic pyelonephritis in our case was xanthogranulomatous pyelonephritis due to the fever, positive U/A, U/C, high ESR, and presence of foamy macrophages [11]; however, our patient had no history of urinary obstruction, and the CT scan lacked the classic bear‐paw appearance. Xanthogranulomatous pyelonephritis in the absence of urinary obstruction is relatively rare but was previously reported in immunocompromised patients [12]. Xanthogranulomatous pyelonephritis is considered a malignancy mimicker and has a wide range of CT appearances [11].

Our case’s immunophenotyping was consistent with previous reports of patients with STK4 deficiency. Immunophenotyping usually shows severe T‐cell lymphopenia and intermittent neutropenia [7]. T‐ and B‐cell lymphopenia is possibly due to the increased apoptosis rate [5, 9]. CD56^+^ cell count does not show a clear pattern, and decreased, normal, and increased cell counts have been reported [7], while low CD16^+^CD56^+^ is seen in such cases [10].

Increased IgG, IgA, and IgE levels, with reduced IgM levels, are seen in these cases. This pattern is also seen in hyper‐IgE syndrome, making it an important differential diagnosis that clinicians must consider [13]. However, very high IgE levels (>1000) are reported to be associated with an atopic phenotype and inborn error of immunity, such as hyper‐IgE and DOCK8 deficiency [14]. Although atopic manifestations correlate with IgE levels, not every patient with elevated IgE demonstrates atopic manifestations [15]. In our case, serum IgE levels were mildly elevated, but the patient had no atopic manifestations. This can result from T‐cell dysregulation, causing the class switching of antibodies to IgE [16].

*STK4* is proposed to play a role in immune function and increase resilience to oxidative stress by interacting with FOXO1 and 3 [2, 7]. Loss of *STK4* leads to immune dysfunction and apoptosis, with the loss of mitochondrial transmembrane potential and enhanced Fas‐mediated apoptosis [7, 10].

Biochemical and structural studies have shown that MST1/STK4 contains, in addition to its N‐terminal kinase domain, C‐terminal regulatory and dimerization regions that modulate MST1 activity and substrate selectivity. Anand et al. [17] demonstrated that the C‐terminal regulatory region and dimerization contribute to MST1 functional activity, including MST1 autophosphorylation within the regulatory region and dimerization‐dependent enhancement of substrate phosphorylation, supporting an important regulatory role for the C‐terminus in MST1 signaling output. At the structural level, the SARAH domain (Sav/RASSF/Hippo) is an α‐helical module in the extreme C‐terminus of MST1 that mediates homodimer formation and heterodimeric interactions with SARAH‐containing partners such as RASSF family proteins and SAV1, providing a mechanistic basis for MST1 complex formation and pathway signaling [18].

Our case involved the c.248del, p.Pro83Leufs ^∗^3 variant, which causes a frameshift in exon 4 of the *STK4* gene. The *STK4* gene is located on chromosome 20q13.12, with the deletion occurring at position 44981829 (GRCh38:NC_000020.11: g.44981829delC). This introduces a premature termination codon well upstream of the last exon, leading to nonsense‐mediated mRNA decay (NMD). The NMD can facilitate mRNA decay, reducing STK4 levels [19], causing the CID phenotype. However, C‐terminal truncating mutations in the last exon evade NMD and lead to the translation of a truncated protein lacking the SARAH domain, critical for STK4’s kinase activity [10]. This shows that structural disruption of functional domains (rather than complete protein loss) can cause CID, albeit with milder phenotypes [10].

This mutation has not been previously documented in ClinVar or HGMD databases. However, pathogenic frameshift variants downstream of the detected variant have been reported. The minor allele frequency of this variant in public population databases (gnomAD, Iranome, and 1000GP) is zero. In silico bioinformatics tools consistently predict a deleterious impact of this variant on the gene and the gene’s products. According to the American College of Medical Genetics (ACMG) guideline, this variant is classified as likely pathogenic. This novel likely pathogenic variant represents a previously unreported cause of CID, and its definite effects on immune function remain to be elucidated.

The management of STK4 deficiency includes the use of antimicrobial agents and immunoglobulin replacement. Hematopoietic stem cell transplant has been successful in some cases [20], but more research is needed about its safety [2]. One important limitation of our study is the inability to analyze the effects of the reported mutation on mRNA or protein; however, this case highlights the importance of considering STK4 deficiency in CID, especially when clinical manifestations are atypical. Our patient’s presentation with chronic pyelonephritis and lymphadenopathy shows how immune dysfunction can lead to complex inflammatory conditions beyond typical infections. Genetic analysis identified a likely pathogenic *STK4* variant, emphasizing the critical role of genetic investigations in resolving ambiguous cases. This report expands our understanding of the phenotypic spectrum of STK4 deficiency and underscores the value of comprehensive diagnostic approaches in identifying underlying immunodeficiencies in patients with atypical presentation.

## Author Contributions

All authors contributed to the study conception, design, and review and editing. Material preparation, data collection, and analysis were performed by Hamidreza Ashayeri, Shabnam Eskandarzadeh, Samin Alavi, Mohammad Saberi, and Azadeh Reshadmanesh. The first draft of the manuscript was written by Hamidreza Ashayeri.

## Funding

The authors received no financial support for the authorship or publication of this article.

## Disclosure

All authors commented on previous versions of the manuscript and read and approved the final manuscript. After using this Grammarly, the authors reviewed and edited the content as needed and take full responsibility for the content of the published article.

## Consent

Written informed consent was obtained from the patient and her parents to publish this case report, including any accompanying images and laboratory results.

## Conflicts of Interest

The authors declare no conflicts of interest.

## Data Availability

All data generated or analyzed during this study are included in this published article.
